# Sub-Lethal Effects of Partially Purified Protein Extracted from *Beauveria bassiana* (Balsamo) and Its Presumptive Role in Tomato (*Lycopersicon esculentum* L.) Defense against Whitefly (*Bemisia tabaci* Genn.)

**DOI:** 10.3390/insects11090574

**Published:** 2020-08-27

**Authors:** Azhar Uddin Keerio, Talha Nazir, Tauqir Anwar, Muhammad Zeeshan Majeed, Yusuf Ali Abdulle, Ghulam Hussain Jatoi, Muswar Ali Gadhi, Dewen Qiu

**Affiliations:** 1 State Key Laboratory for Biology of Plant Diseases and Insect Pests, Institute of Plant Protection, Chinese Academy of Agricultural Sciences, Beijing 100081, China; talha23december@gmail.com (T.N.); idaajaa236@gmail.com (Y.A.A.); jatoighulamhussain@hotmail.com (G.H.J.); muswarali860@gmail.com (M.A.G.); 2 Pest Warning & Quality Control of Pesticides, Punjab Agriculture Department, Government of the Punjab, Sillanwali 40010, Pakistan; tauqeer26@gmail.com; 3 Department of Entomology, University of Sargodha, Sargodha 40100, Pakistan; zeeshan.majeed@uos.edu.pk; 4 Department of Plant Pathology, Sindh Agriculture University, Tandojam 70060, Pakistan

**Keywords:** *Beauveria bassiana*, *Bemisia tabaci*, fungal proteins, induced resistance, survival, fecundity, salicylic acid pathway, jasmonic acid pathway

## Abstract

**Simple Summary:**

Apart from their direct entomopathogenicity, many entomopathogenic fungi synthesize protein molecules that can trigger plant defense mechanisms against herbivore insect pests. This laboratory study determined the sub-lethal effects of a partially purified protein derived from *Beauveria bassiana* against whitefly *Bemisia tabaci* on tomato plants along with the subsequent gene expression analyses of key gens potentially linked to jasmonic acid (JA) and salicylic acid (SA) associated plant defense pathways. The exogenous foliar application of *B. bassiana*-derived protein significantly reduced the whitefly survival and fecundity parameters concomitantly with an up-regulation of all the plant defense associated genes, particularly of SA pathway genes. These findings demonstrate the putative role of this partially purified entomopathogenic fungal protein and suggest its further purification and characterization for using in future microbial pest control strategies against whiteflies and other sap-feeding insect pests.

**Abstract:**

Plants rely on various physiological and molecular defense mechanisms against biotic stresses such as herbivore insects. Many entomopathogenic fungi synthesize protein molecules that can trigger these plant defenses. This laboratory study characterized the bioactivity of a partially purified protein derived from *Beauveria bassiana* (ARSEF 2860) against whitefly, *Bemisia tabaci* (Hemiptera: Aleyrodidae), which is an economically important pest of agricultural and horticultural crops worldwide. Different concentrations (i.e., 0.021, 0.042 and 0.063 μM) of fungal protein were bioassayed to determine their sub-lethal effect on the survival percentage and fecundity rate of *B. tabaci* on tomato (*Lycopersicon esculentum*) plants. In addition, the putative role of this partially purified *B. bassiana* protein in the defense mechanisms of plant was assessed through the expression analyses of important genes related to salicylic acid (SA)—and jasmonic acid (JA)—associated pathways using RT-qPCR. Results revealed a significant suppression of the survival percentage and fecundity rate of *B. tabaci* by the fungal protein. Lowest survival (41%) was recorded for the highest concentration of protein (0.063 μM), whereas mean survival for the other two protein concentrations (0.042 and 0.021 μM) were 62 and 71%, respectively. Likewise, the highest and lowest mean fecundity rates were observed for the control and the highest protein concentration (i.e., 3.3 and 1.8 eggs day^−1^ female^−1^, respectively). Furthermore, the exogenous application of *B. bassiana*-derived protein on tomato plants strongly up-regulated the SA-related genes (PAL, PR1, BGL2 and EDS1) and slightly up-regulated the JA-related genes (AOC, AOS, OPR3 and LOX) as compared to the control plants. These findings demonstrate the putative role of this partially purified *B. bassiana* protein fraction in inducing systemic resistance in the tomato plants against *B. tabaci*, suggesting its further purification and characterization to be used as novel biological pest control tool against *B. tabaci* and other sap-sucking insect pests.

## 1. Introduction

Whiteflies (Aleyrodidae: Hemiptera) are one of the most destructive sucking pests of field and horticultural crops and ornamental plants around the globe [1]. These insect pests cause substantial economic losses directly by sap-feeding of host plants and indirectly by causing different physiological disorders such as the interruption of normal plant photosynthetic activity by sooty mold growth on the host plants mediated by whitefly honeydew secretions. Furthermore, whiteflies transmit more than 114 species of plant viruses, many of which cause economically important plant diseases such as tomato yellow leaf curl virus (TYLCV), cotton leaf curl virus (CLVC), cucumber vein yellowing virus (CVYV), etc. [1,2,3].

The whitefly, *Bemisia tabaci* Genn., is one of the most invasive species worldwide. Farmers rely exclusively on the application of synthetic insecticides, particularly of organochlorines and pyrethroids, to combat *B. tabaci* infestations [4,5,6]. The extensive use of synthetic chemicals against whitefly populations has let to various problems such as environmental contamination, disruption of non-target fauna, pest resistance and resurgence and human health hazards. Moreover, owing to high genetic diversity, rapid population growth rates and obscure feeding behavior, whitefly populations have developed resistance against a wide range of synthetic insecticides belonging to all major chemical classes [5,6,7,8,9]. This situation necessitates seeking out alternate pest control methods that are environment-friendly and biorational. Microbial biopesticides, for instance, are one of the emerging and promising biological pest control strategies being developed and evaluated against many insect pest species, including *B. tabaci* [10,11,12,13,14].

Entomopathogenic fungi (EPFs) are one of the important microbial pest control agents which have been extensively studied and proven effective against a broad range of insect pests [15,16]. Due to their low mammalian toxicity and high host specificity, EPFs have been a core area in contemporary biological control research [17,18]. Apart from their direct entomopathogenicity, EPFs produce a variety of secondary metabolites which can be harmful to many insect pests [19]. For example, destruxins, efrapeptins and cordycepins, secreted respectively by *Metarhizium anisopliae*, *Tolypocladium* sp. and *Cordyceps militaris*, revealed the insecticidal and antifeedant effects against different lepidopterous pests [20,21]. Similarly, the crude proteins derived from *M. anisopliae*, *B. bassiana* and *Isaria fumosorosea* caused significant mortality of *Musca domestica* [22] and *Spodoptera littoralis* [23]. Recently, authored in [24,25] evidenced the toxicity of filtrates and conidia extracted from different isolates of *Lecanicillium lecanii* and *B. bassiana* against *M. persicae*.

Moreover, some proteins synthesized by many necrotrophic and biotrophic fungi can induce resistance in plants to pathogens and herbivore pests [26,27,28]. Indeed, EPFs have the capability to develop endophytically within different plant tissues and induce systemic resistance in the host plants against various biotic stresses including nematodes, pathogens and phytophagous insects [29,30]. They have also been shown to increase the yield and growth of the plants [31,32]. This attained resistance is commonly manifested by developmental, physiological and/or biochemical responses of the plants under attack to any stress stimuli, for example insect herbivory, and is usually triggered through different plant defense response and signaling pathways [33].

Salicylic acid (SA) and jasmonic acid (JA) are important signaling molecules involved in the activation of plant defense pathways [33,34]. Although both of these plant defense pathways are triggered accumulatively in response to herbivores attack, the response of JA-associated pathway is predominantly linked to chewing herbivores [35,36], while the response of the SA-associated pathway is linked to the phloem-sucking insect pests such as aphids and whiteflies [26,37,38].

Keeping in view the above mentioned novel biological mechanisms of EPFs and their secondary proteins in the management of insect pests, this study was aimed to determine the bioactivity of a partially purified protein derived from *B. bassiana* (ARSEF 2860) against *B. tabaci* on tomato (*Lycopersicon esculentum* L.) plants. Furthermore, the expression levels of key genes potentially associated with JA and SA pathways were determined using RT-qPCR in order to assess the putative role of this partially purified EPF protein in induced plant resistance against *B. tabaci*.

## 2. Materials and Methods

### 2.1. Plant Culture and Rearing of B. tabaci

The seeds of tomato (*L. esculentum*) were soaked for 7–10 days in a sterilized Petri-plate (100 × 15 mm) on a moistened filter paper disc. The healthy, vigorous and uniform tomato seedlings were transferred individually to the plastic pots (100 × 150 mm) filled with a sterile mix of soil. The pots containing tomato seedlings were then kept in the growth chamber at a controlled temperature (27 ± 2 °C (day) and 18 ± 2 °C (night)) and relative humidity (60–70%). The population of *B. tabaci* (having mixed male and female individuals) was collected from the greenhouse maintained tomato plants (CAAS, Beijing, China) and was shifted and reared on the tomato plants in laboratory at 55–65% relative humidity and 25 ± 2 °C temperature with a 16:8 h (L:D) photoperiod for about three months.

### 2.2. Culture of B. bassiana

The isolated spores of *B. bassiana* (ARSEF 2860), preserved in 20% glycerol at −80 °C, were acquired from the State Key Laboratory for Biology of Plant Diseases and Insect Pests of the Institute of Plant Protection (CAAS, Beijing, China). These fungal spores were grown on potato dextrose agar (PDA) medium (potato 200 g/L, dextrose 20 g/L and agar 20 g/L) in sterilized polystyrene Petri-plates (100 × 15 mm) at 25 ± 2 °C for 25 days in the dark.

### 2.3. Preparation of Crude Protein and Its Partial Purification

**Figure 1:**
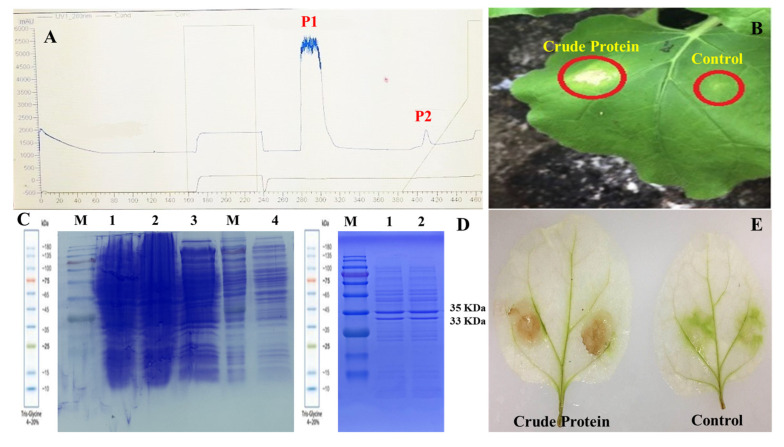
Crude protein purification from *B. bassiana* (ARSEF 2860). (**A**) Ion-exchange chromatography exhibited two peaks (1 and 2) of crude protein acquired from 80% ammonium sulfate precipitation and purified by the ÄKTA Explorer 10 protein purification system; (**B**) the necrosis induced by crude protein (0.063 μM) in the leaves of *N. tabacum* observed at 24 h post treatment. In control treatment, the leaves of *N. tabacum* were treated with buffer (50 mM Tris-HCL, pH 8.0); (**C**) filtrate (lanes 1, 2 and 3) and proteins (lane 4) derived from *B. bassiana* precipitated on 12% SDS-PAGE using 80% ammonium sulfate. Lane M corresponds to molecular weight of protein markers; (**D**) *B. bassiana*-derived proteins purified through chromatography column resolved on 12% SDS-PAGE. Lane M corresponds to molecular weight of protein markers, shown in kilodaltons. (**E**) ROS burst in the cells of *N. tabacum* observed after crude protein treatment. Brown DAB blemished precipitates represent the ROS burst.

For this purpose, a previously described protocol [39] was followed. In brief, primary culture of *B. bassiana* was prepared by adding 2 mL of conidial suspension (1.0 × 10^8^ conidia mL^−1^) in 100 mL of potato dextrose broth (PDB: dextrose 20 g/L and potatoes 200 g/L) for 2 days on a rotatory shaker at 150 rpm and 24 °C. Then, the secondary culture was prepared by the addition of 20 mL primary culture in 2 L of potato dextrose broth for 6 days on a rotatory shaker at 180 rpm and 24 °C. Secondary culture was then centrifuged at 4 °C for 30 min at 12,000 rpm, and the resultant supernatant was filtered via a 0.45 μm filter (Millipore Corp., Billerica, MA, USA). Partial protein purification was done using an ÄKTA Explorer 10 protein purification system (GE Healthcare, Piscataway, NJ, USA) using a HiTrap^TM^ HP Q 5 mL chromatography column (GE Healthcare, Uppsala, Sweden). Column equilibration was done by buffer A (50 mM Tris-HCl; pH 8.0), and the sample of crude protein was loaded at a flow rate of 2 mL min^−1^. The column was washed again with buffer A. Afterwards, the buffer B (50 mM Tris-HCl; 1 M NaCl pH 8.0) was used to elute the proteins bounded in the column at a flowrate of 2 mL/min. A desalting column (GE Healthcare, Uppsala, Sweden) was used to desalt the eluted protein fraction. The desalted fraction of partially purified protein was analyzed using 12% sodium dodecyl sulfate polyacrylamide gel electrophoresis (SDS-PAGE). The fraction contained two proteins of 33 and 35 kDa (Figure 1). This partially purified protein was stored at −20 °C until its application in downstream bioassays. The protein concentrations were determined using a BCA protein assay kit (Pierce, Rockford, IL, USA).

### 2.4. Bioassay of Protein Activity against B. tabaci

The bioactivity of *B. bassiana*-derived partially purified protein was determined against *B. tabaci* whitefly in laboratory bioassays. Treatments used in these bioassays included three protein concentrations (i.e., 0.021, 0.042 and 0.063 μM) and the control. Laboratory-maintained potted tomato (*L. esculentum*) plants at the 5-leaf stage were used in these bioassays. Four milliliters of each protein and/or control treatment solutions, prepared using buffer (50 mM Tris-HCl; pH 8.0), were sprayed on each plant using an aerosol spray bottle, and the sprayed plants were left to dry at room temperature (25 °C) for 24 h. In order to determine the survival potential of *B. tabaci*, 10 healthy and active young whiteflies (with a 1:1 male to female ratio) were collected from laboratory-maintained *B. tabaci* culture and were confined on plant leaves using small leaf clip-cages (made up of transparent polystyrene rims (25 × 15 mm) fitted with a fine fabric mesh on one side). Mortality of these whitefly adults was recorded daily after every 3 h for eight consecutive days. In order to find out the fecundity rate of *B. tabaci*, one pair of healthy and active young whitefly adults per plant was confined in leaf clip-cages. The number of eggs per female whitefly adult laid in these leaf clip-cages was noted for eight consecutive days. Ten independent replications were maintained for each treatment. All bioassays were performed at 25 ± 2 °C and 55–65% relative humidity.

### 2.5. RNA Extraction and cDNA Synthesis

For the molecular characterization of putative plant resistance against *B. tabaci* induced by *B. bassiana*-derived protein, tomato plants were sprayed with the highest protein concentration (i.e., 0.063 μM), and after 24 h, these treated plants were infested by *B. tabaci* adults. At 24, 48, 72 and 92 h post-insect exposure, three leaves were randomly taken from each of the three protein-treated and/or control (buffer-treated) plants, and their total RNA was extracted using the TransGen EasyPure^®^ plant RNA kit (TransGen Biotech, Beijing, China) following the manufacturer’s protocol. Extracted RNA was quantified using a NanoPhotometer^®^ (NP80 Touch, Implen Inc., Foster, CA, USA). Reverse transcription was then carried out to synthesize cDNA using a TransScript^®^ One-Step gDNA Removal and cDNA Synthesis Super Mix kit (TransGen Biotech, Beijing, China).

### 2.6. Real-Time Quantitative PCR (RT-qPCR)

Relative expression of the key genes possibly involved in the defense mechanisms of *L. esculentum* plants was determined in whitefly-infested protein treated (with 0.063 μM) and control (buffer-treated) plants through RT-qPCR. The tested key genes were PAL, PR1, BGL2 and EDS1 of the SA pathway and AOC, AOS, OPR3 and LOX of the JA pathway. β-actin was used as an internal control (reference gene: accession no. U60481.1). Primer pairs used for the amplification of these plant defense genes are detailed in Table S1. For RT-qPCR amplifications, the thermocycler ABI 7500 Real-Time PCR System (Applied Biosystems, Foster, CA, USA) was used. The reaction mixture contained 20 μL of PCR mixture (10 μL 2 × SYBR^®^ Premix Ex Taq (Takara, Dalian, China), 7 μL ddH_2_O, 2 μL cDNA template and 0.5 μL of reverse and 0.5 μL of forward primer). The thermal protocol followed was the same as described previously [39], including a preheating at 95 °C for 30 s, denaturation at 95 °C for 30 s (40 cycles), annealing at 60 °C for 40 s and elongation at 72 °C for 60 s. There were a total nine biological replicates (three leaves taken from each of the three independently treated tomato plants) for each treatment. The qPCR amplifications were repeated three times, and the expression levels were set at 1.0 at day 0 for each gene.

### 2.7. Statistical Analysis

Statistical analysis of the data was carried out using SPSS version 20.0.0 (SPSS Inc., Chicago, IL, USA). All experiments were repeated three times independently, and the mean values of all parameters are presented in figures along with the standard errors. The significant differences among treatments were determined using univariate general linear model of analysis of variance (ANOVA), followed by Student–Newman–Keuls (SNK) at a 0.05 level of probability. RT-qPCR expression levels of SA- and JA-associated genes were determined using the comparative *C*_T_ method. In brief, the Δ*C*_T_ value was obtained by subtracting the *C*_T_ value of reference gene from the *C*_T_ value of target gene. The normalized fold changes of the expression of target genes were expressed as 2^−ΔΔCT^ (=Δ*C*_T_ treated sample − Δ*C*_T_ control).

## 3. Results

### 3.1. Crude Protein-Induced Necrosis in Tobacco Leaves

Ion-exchange chromatography of partially purified protein extracted from *B. bassiana* (ARSEF 2860) exhibited two peaks (Figure 1A). These two protein peaks were collected, desalted and infiltered into the leaves of tobacco (*Nicotiana tabacum* cv. Samsun-NN) to find out their bioactivity. Prominent necrotic zones appeared on the treated leaf areas 24 h post-infiltration (Figure 1B). Afterward, SDS-PAGE gel analysis produced two distinct protein bands of molecular weight of 33 and 35 kDa (Figure 1D). These protein bands were recovered and further bioassayed for their necrosis-inducing activity. Considerable reactive oxygen species (ROS) burst was observed in the leaves of *N. tabacum* treated with *B. bassiana*-derived crude protein, while no ROS burst was produced in the leaves treated with buffer (control) (Figure 1E).

### 3.2. Effect of B. bassiana-Derived Protein on B. tabaci Survival

**Figure 2:**
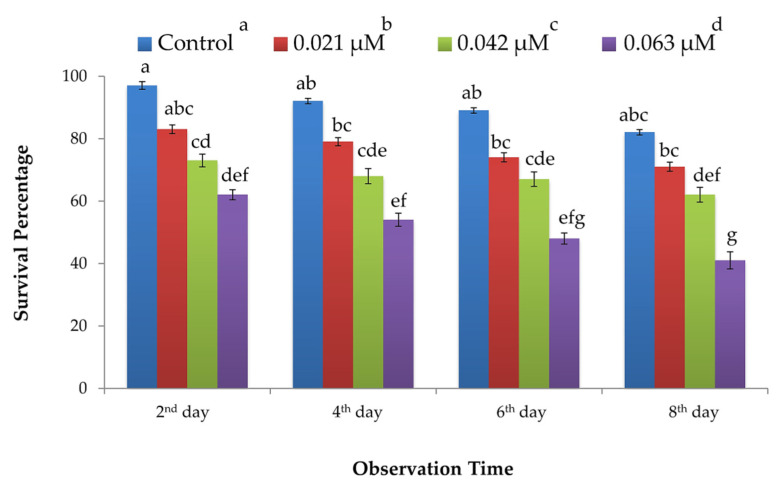
Survival percentage (± SEM; *n* = 10 for each treatment) of whitefly (*B. tabaci*) recorded for different concentrations of partially purified protein derived from *B. bassiana* (ARSEF 2860). Different letters indicate significant difference among treatments (univariate ANOVA; SNK test at α = 0.05). (cf. Table 1).

Results of protein activity bioassays showed that the overall survival percentage of *B. tabaci* was significantly reduced on the tomato plants treated with *B. bassiana*-derived protein as compared to the insect survival on buffer-treated (control) plants. There was a significant effect of different concentrations of fungal protein (F_3, 144_ = 37.99; *p* ≤ 0.001) and time intervals (F_3, 144_ = 5.29; *p* ≤ 0.001) on the mean survival of *B. tabaci*, whereas the interaction of factors (i.e., concentrations and time) had a non-significant impact (F_9, 144_ = 0.15; *p* = 0.998) (See Table 1). Whitefly survival percentage was reduced along with the concentration of fungal protein. Lowest survival (41–62%) was recorded for the highest concentration of protein (0.063 μM), whereas the highest survival (82–97%) was observed for the buffer-treated (control) plants. Mean survival of *B. tabaci* for the other two protein concentrations (i.e., 0.021 and 0.042 μM) were 71 and 62%, respectively (cf. Figure 2).

### 3.3. Effect of B. bassiana-Derived Protein on B. tabaci Fecundity

**Figure 3:**
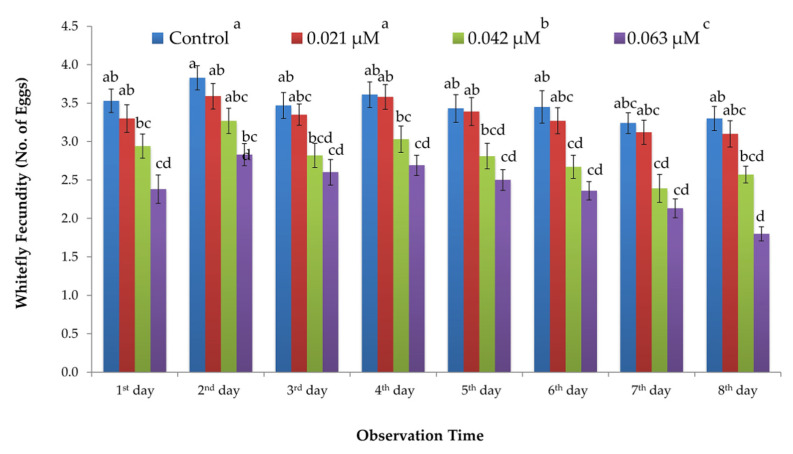
Fecundity rate (±SEM; *n* = 5 for each treatment) of whitefly (*B. tabaci*) recorded for different concentrations of partially purified protein derived from *B. bassiana* (ARSEF 2860). Different letters indicate significant difference among treatments (univariate ANOVA; SNK test at α = 0.05). (cf. Table 2).

Similar trend was recorded for the impact of *B. bassiana*-derived protein on the fecundity rate of *B. tabaci*. Fecundity was significantly reduced on protein-treated plants as compared to control ones. A significant effect of protein concentrations was recorded on the fecundity of *B. tabaci* (F_3, 288_ = 7.52; *p* ≤ 0.001), whereas time alone and the interaction of concentration and time showed a non-significant impact (Table 2). Mean fecundity of *B. tabaci* was reduced along with the protein concentrations. The lowest fecundity rate (1.8 eggs day^−1^ female^−1^) was observed for the highest concentration (0.063 μM) of protein, while the maximum fecundity rate (3.1 eggs day^−1^ female^−1^) was recorded for the lowest concentration (0.021 μM) of protein. The mean fecundity rate observed for 0.042 μM concentration of protein was 2.3 eggs day^−1^ female^−1^. Buffer-treated (control) plants exhibited the maximum fecundity rate (3.3 eggs day^−1^ female^−1^) (cf. Figure 3).

### 3.4. Expression Levels of Plant Defense Related Genes in Response to B. basiana-Derived Protein

**Figure 4:**
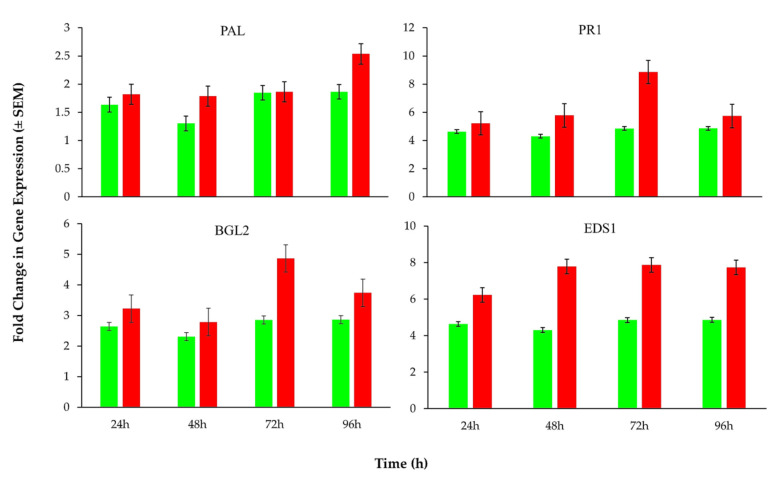
Expression levels of key genes potentially linked to the salicylic acid (SA)-associated plant defense pathways determined at different post-treatment time intervals (sample size *n* = 9 for each treatment). Green and red columns represent the results for buffer-treated (control) and protein-treated plants, respectively.

**Figure 5:**
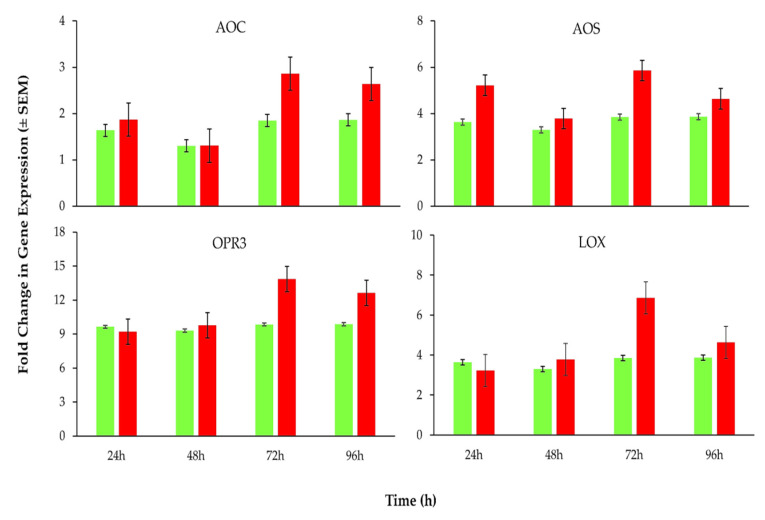
Expression levels of key genes potentially linked to the jasmonic acid (JA)-associated plant defense pathways determined at different post-treatment time intervals (sample size *n* = 9 for each treatment). Green and red columns represent the results for buffer-treated (control) and protein-treated plants, respectively.

**Figure 6:**
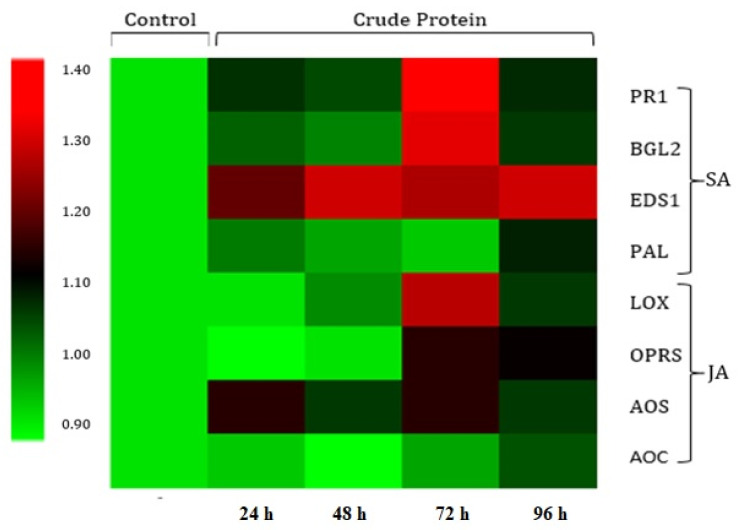
Heat map for genes expression linked to the JA- and SA-associated plant defense pathways showing the relative expression levels at 24, 48, 72 and 96 h post-insect exposure. Scale color bar in the picture shows low (green) to high (red) gene expression. The heat map was generated using Multi Experiment Viewer (MeV, version 4.6.0) software.

In order to find out the putative role of *B. bassiana*-derived protein in induced resistance in *L. esculentum* plants against *B. tabaci*, the expression of key genes related to JA- and SA-associated plant defense pathways were analyzed by RT-qPCR. Results revealed a significant (*p* = 0.05) up-regulation of all SA-associated pathway genes (PAL, PR1, BGL2 and EDS1; Table S1) observed at all post-insect exposure time intervals (Figure 4), whereas JA-associated pathway genes (AOC, AOS, OPR3 and LOX; Table S1) were moderately or slightly up-regulated (Figure 5). The maximum expressions of all the tested genes were recorded at 72 h post-insect exposure, as evidenced by a heat map of the gene expressions (Figure 6).

## 4. Discussion

Plants rely on an array of defense mechanisms regarding their response to various biotic stresses, for example insect herbivory [40,41]. These defense mechanisms are usually activated by different types of signaling molecules such as SA and JA, and through several microbe-induced and herbivore-induced elicitor molecules [34]. These elicitors are often glycoproteins, proteins and lipids, which trigger resistance in the plants against different pathogens and herbivores [42,43,44]. Similarly, some strains of EPFs synthesize certain protein molecules in addition to their direct entomotoxicity, which can induce plant resistance against different pathogens and herbivore insects [27].

This laboratory study evaluated the bioactivity of a partially purified protein derived from EPF *B. bassiana* (ARSEF 2860) against whitefly *B. tabaci* on tomato (*L. esculentum*) plants. Plants treated with the exogenous application of *B. bassiana* protein manifested a significant suppression of the survival and fecundity of *B. tabaci* as compared to the control plants. These findings are in accordance with [45], which showed a significant reduction in the fecundity of *Aphis gossypii* by the exogenous applications of fungal filtrates and conidial of endophytic fungi *B. bassiana* and *L. lecanii*. Likewise, our findings corroborate the results of [22] demonstrating a considerable negative impact of the crude protein derived from *B. bassiana*, *I. fumosorosea* and *M. anisopliae* on the survival percentage of *M. domestica*. Our results are in accordance as well with authors in [39], who revealed that feeding on plants of *L. esculentum* treated by *L. lecanii*-derived partially purified protein significantly affected the fecundity rate and survival percentage of *M. persicae*. Similarly, some previous studies have revealed the significant sub-lethal and toxic effects of crude proteins extracted from different isolates of *M. anisopliae* and *B. bassiana* on *Dysdercus cingulatus* [46] and *S. litturalis* [23]. Moreover, some previous works proved that the exogenous application of such proteins related to plant defense as jasmonates and methyl salicylate might disrupt the activity of chewing [47] and sucking [48,49] insect pests on tomato plants.

Gene expression analyses showed that the exogenous application of *B. bassiana*-derived protein strongly up-regulated the SA-associated pathway genes, while those related to JA-associated pathway were partially up-regulated. These results validate the fact that herbivory by sap-sucking insects, for instance whiteflies, is more often manifested with enhanced expression levels of SA pathway-related genes [34,38]. Our results are in line with a recent study [50] that revealed important sub-lethal effects of the *B. bassiana*-derived PeBb1 protein elicitor on *M. persicae* on the plants of *Brassica rapa* concomitantly with the enhanced expression of JA and Et pathway-related genes.

The results of this study elucidate the potential eliciting role of *B. bassiana*-derived protein in suppressing *B. tabaci* fecundity rate and survival percentage on the plants of tomato. However, as different plant elicitor molecules, such as SA and JA, may activate the inhibition of proteinase in tomato plants [47,51], additional studies are required to better understand how this *B. bassiana*-derived protein influences the plant systems and insect fitness parameters. Moreover, although this fungal protein showed significant suppression of *B. tabaci* life traits under laboratory conditions, however, keeping in view the high biotic potential and population growth rate of *B. tabaci*, further studies are needed to enhance the anti-insect effect of this protein, so that it can be used successfully as an important biological pest management tool under field conditions.

## 5. Conclusions

In brief, this laboratory study demonstrated that feeding on *L. esculentum* plants treated with *B. bassiana*-derived partially purified protein significantly reduced the fecundity rate and survival percentage of *B. tabaci*. Furthermore, the expression level of key genes associated with plant defense signaling pathways were highly up-regulated in the plants receiving the exogenous application of *B. bassiana*-derived protein, recommending its putative efficiency as novel biocontrol tool against *B. tabaci* and other sap-feeding herbivores. However, further studies are needed regarding the purification and functional and molecular characterizations of this partially purified *B. bassiana* protein.

## Figures and Tables

**Table 1:** Univariate analysis of variance table for the effect of partially purified protein derived from *B. bassiana* (ARSEF 2860) on the survival percentage of *B. tabaci* (cf. Figure 2).

SOV	DF	Type III SS	MS	F-Value	*p*-Value *
Concentration	3	32,206.9	10,735.6	37.99	<0.001
Time	3	4486.9	1496.5	5.29	<0.001
Concentration × Time	9	385.6	42.8	0.15	0.9980
Error	144	40,690.0	282.6		
Total	159	77,769.4			

* *p* < 0.001 (highly significant) and *p* < 0.05 (significant); univariate analysis of variance (UNIANOVA) at α = 0.05; DF: degrees of freedom; SS: sum of squares; MS: mean sum of squares; F: F-statistic; CV: coefficient of variation; GM: grand mean.

**Table 2:** Univariate analysis of variance table for the effect of partially purified protein purified of *Beauveria bassiana* (ARSEF 2860) on the fecundity rate of *B. tabaci* (cf. Figure 3).

SOV	DF	Type III SS	MS	F-Value	*p*-Value *
Concentration	3	59.261	19.7538	7.52	<0.001
Time	7	14.162	2.0231	0.77	0.6130
Concentration × Time	21	2.882	0.1373	0.05	1.0000
Error	288	776.115	2.6278		
Total	391	850.966			
GM/CV	3.02/53.75				

* *p* < 0.001 (highly significant) and *p* < 0.05 (significant); univariate analysis of variance (UNIANOVA) at α = 0.05; DF: degrees of freedom; SS: sum of squares; MS: mean sum of squares; F: F-statistic; CV: coefficient of variation; GM: grand mean.

## Supplementary Materials

The following are available online at https://www.mdpi.com/2075-4450/11/9/574/s1, Table S1: Primer pairs used in this study for RT-qPCR amplifications of the key genes potentially involved in the plant defense (SA and JA) pathways.
