# Supplementary material for: Sub-Lethal Effects of Partially Purified Protein Extracted from Beauveria bassiana (Balsamo) and Its Presumptive Role in Tomato (Lycopersicon esculentum L.) Defense against Whitefly (Bemisia tabaci Genn.)
Source: Insects. 2020 Aug 27;11(9):574. doi: 10.3390/insects11090574 (PMC7564989; doi:10.3390/insects11090574)
Supplement: Supplementary file 1 [file insects-11-00574-s001.pdf]

Supplementary Tables

Table 1. Primer pairs used in this study for RT-qPCR amplifications of the key genes potentially involved in the plant defense (SA and JA) pathways.

Gene	Description	Primer sequence
β-ACTIN	Housekeeping gene	F: 5′-GGAAAATCAGTCTCGGTTCAG-3′
		R: 5′-TCATACAGCAGCAAGCAC-3′
AOC	JA-regulated	F: 5′-AGCCCTGCTTATCTCGATT-3′
		R: 5′-ATCCATTCAAGCAAGTAT-3
AOS	JA-regulated	F: 5′-TCTTGAAATTCAAATATCA-3′
		R: 5′-ATCCATTCAAGCAAGTAT-3′
BGL2	JA-regulated	F: 5′- CACCAACATTCACATAACAGAGGC-3′
		R: 5′-AGGGCTGATTTCATTACCAAC-3′
LOX	JA-regulated	F: 5′- AGAGATGTGGAATTAGCTCG-3′
		R: 5′-AAGGAACTAGGTATTTCATGT-3′
PAL	SA-regulated	F: 5′- TTCAAGGCTACTCTGGC-3′
		R: 5′-CAAGCCATTGTGGAGAT-3′
PR1	SA-regulated	F: 5′- ATCTCATTGTTACTCACTTGTC-3′
		R: 5′-CAAGCCATTGTGGAGAT-3′
OPR3	SA-regulated	F: 5′-GGACGCAACTGATTCTGACCCAC-3′
		R: 5′-CGTAGGCGTGGTAGCGAGGTTG-3′
EDS1	SA-regulated	F: 5′-CGAAGGGGACATAGATTGGA-3′
		R: 5′-ATGTACGGCCCTGTGTCTTC-3′
